# Psycho-Emotional Impact of Anomaly Ultrasound Scan in Romanian Pregnant Women

**DOI:** 10.3390/healthcare9111415

**Published:** 2021-10-21

**Authors:** Liana Pleş, Bashar Haj Hamoud, Mihai Cornel Traian Dimitriu, Cătălin Cîrstoveanu, Bogdan Socea, Antoniu-Crîngu Ionescu, Dragoş Albu, Romina-Marina Sima, Mircea-Octavian Poenaru

**Affiliations:** 1 Department of Obstetrics and Gynecology, “Carol Davila” University of Medicine and Pharmacy, 020021 Bucharest, Romania; liana.ples@umfcd.ro (L.P.); mihai.dimitriu@umfcd.ro (M.C.T.D.); cringu.ionescu@umfcd.ro (A.-C.I.); dragos.albu@umfcd.ro (D.A.); romina.sima@umfcd.ro (R.-M.S.); mircea.poenaru@umfcd.ro (M.-O.P.); 2 Department of Obstetrics and Gynecology, “Sf. Ioan” Hospital—Bucur Maternity, 040294 Bucharest, Romania; 3 Department for Gynecology, Obstetrics and Reproductive Medicine, Saarland University Hospital, 66421 Homburg, Germany; bashar.hajhamoud@uks.eu; 4 Department of Obstetrics and Gynecology, “Sf. Pantelimon” Emergency Hospital, 021659 Bucharest, Romania; 5 Department of Pediatrics, “Carol Davila” University of Medicine and Pharmacy, 020021 Bucharest, Romania; catalin.cirstoveanu@umfcd.ro; 6 Pediatrics Department, “Maria Sklodowska Curie” Emergency Children Clinical Hospital, 041451 Bucharest, Romania; 7 Department of Surgery, “Carol Davila” University of Medicine and Pharmacy, 020021 Bucharest, Romania; 8 Department of Surgery, “Sf. Pantelimon” Emergency Hospital, 021659 Bucharest, Romania

**Keywords:** anomaly scan, maternal anxiety, anxiety scale, prenatal psychological screening, Romania

## Abstract

Objective: Second-trimester anomaly scan was introduced as a regulated practice in Romania in 2019, causing misperceptions and unrealistic expectations about this examination among pregnant women. This study aimed to assess whether second trimester anomaly scan is a reason “per se” for maternal anxiety. Design: A prospective type 1 cohort study was conducted in a tertiary prenatal diagnosis center with three locations in Bucharest, Romania, among pregnant women who underwent a second trimester anomaly scan between 1 December 2019 and 29 February 2020. Main outcome measure: Anxiety at the time of prenatal anomaly scan. Results: Out of the 138 participants, 32.6% believed that the anomaly scan could detect all fetus defects, 13.8% considered that the baby is bothered by the probe “light”, 8.7% believed that the scan could harm the fetus, 96.4% reported that it was a pleasant experience, and 95% felt that it strengthened their bond with the fetus. The State-Trait Anxiety Inventory (STAI) score revealed that women with high state anxiety were more anxious at pre-scan (*p* = 0.001). Conclusion: Ultrasound scan in the second trimester is correlated with a significant anxiety for women who are prone to this psychological trait. It is also a good opportunity to screen for highly anxious women who could benefit from prenatal psychological counseling to facilitate timely recognition and prevention of postpartum psychiatric disorders such as depression.

## 1. Introduction

Ultrasonography represents a relatively cheap, easily reproducible, repeatable, and no harmful diagnostic investigation. It gathered new valences in recent times, such as point of care ultrasound (POCUS), pulmonary ultrasound, and others [1]. The importance of ultrasound in pregnancy has been increasing lately, starting from the first trimester till the end of pregnancy [2]. There are many studies published worldwide concerning the psychological impact of the ultrasound and prenatal procedures on maternal psychological status [3,4]. These studies were conducted in the time when ultrasound were mainly in 2D and had less psychological impact than the recent technologies as 3D and 4D and are mainly directed towards the diagnosis procedures, i.e., amniocentesis and the feedback of the scan.

In Romania, the second trimester anomaly scan was introduced as a regulated practice (by national guidelines) only in 2019, although it had been extensively used for more than 15 years before that. Under these circumstances, various misperceptions and unrealistic expectations towards this examination arose among pregnant women. The impact of this crucial examination on the psycho-emotional status of women and couples has been neglected and it has raised several issues that are not currently addressed by physicians. Among these, the main issues are: “What are the purposes and limits of the examination?”, “Is the scan safe for the baby?”, “Which information sources are women accessing prior to the ultrasound scan?”, “Does the anomaly scan induce anxiety among all women or are some women more prone to feeling anxious?”, “Are there any psychological benefits of the scan concerning the bonding between the mother and the fetus?”, “How important is the ‘entertainment’ dimension of the procedure?”, and “Can the patient–physician relationship and counseling be improved via the experience of this procedure?”

Several studies focusing on prenatal anxiety have attempted to validate measures for assessing it; however, in Romania, this topic is underestimated and often overlooked, and this is why we intended to bring it into the health care providers’ attention. This study aims to identify the specific issues in the national context to provide effective counseling and reduce anxiety and stress related to prenatal procedures [5].

## 2. Material and Methods

A prospective type 1 cohort study was conducted in a three-location tertiary prenatal diagnostic center in Bucharest, Romania, among pregnant women who underwent a second trimester anomaly scan between 1 December 2019 and 29 February 2020 (according to national protocols ultrasound scan for fetal anomalies between 20 and 23 weeks of gestation). The study was approved by the Ethical Committee and informed consent was obtained from each participant.

The main objective of the study was to assess if the second trimester anomaly scan is a reason “per se” for maternal anxiety. Secondary objectives were to identify the conditions that can influence maternal anxiety and their impact on counseling, the psycho-emotional issues that could be improved while performing the scan, and the ways in which the women could be educated to be more realistic in their expectations regarding the scan.

The inclusion criteria were: mid trimester pregnancy, anomaly-scan ultrasound, and agreement to participate. Women who were scheduled for recheck, presented for biometry, could not read, or did not complete all the sections of the questionnaire were excluded from the analysis.

Women who agreed to participate received a questionnaire prior to the scan and were informed about the aim of the study, the anonymity of the participation, and the correct way to fill the questionnaire. The participants were required to complete the first two sections before the scan and the third section after it on paper. The questionnaire is attached in Appendix A.

The three-section questionnaire used in this study is not a standardized tool and was constructed by our team. The first section included questions about participant’s age, previous experience with pregnancy and ultrasound scans, level of education, and social environment to allow studying the possible relationships between these socio-demographic factors and the outcome variable. The other items in this section were related to the participants’ source of information about anomaly ultrasounds; the familial, social, and “entertainment” aspects of the scan; and the anxiety experienced by the participants before the scan. The final six items of this section were directly related to the anomaly scan and each item measured the participant’s pre-scan anxiety on a 4-point Likert-type scale, ranging from “not at all” [1] to “very much so” [6].

The second section of the questionnaire aimed to evaluate anxiety as a feeling being experienced at the moment of the examination, and utilized the State-Trait Anxiety Inventory (STAI) Form Y-1, comprising 20 items (e.g., “I feel calm”, “I feel tense”) measured on a 4-point Likert-type scale ranging from “not at all”, equivalent for one point, to “very much so”< equivalent for 4 points [4]. A cut-off point of 40 was used as suggested by Grant et al. (2008), who used STAI with a perinatal population [7].

A reasonable time (approximately 20 min) was allocated to the participants to fill the questionnaire so that they could work at a relaxed pace without feeling any pressure, thereby keeping a check on scan-related anxiety and anticipation.

The third section of the questionnaire had to be filled after the scan and comprised questions regarding the participants’ experience of the scan (e.g., “Did you get the information you wanted about the baby?”).

The responses collected were entered into a database file and statistical analyses were performed using SPSS program (version 25.0, IBM, Armonk, NY, USA). Pearson’s correlation coefficients were used and considered statistically significant for *p* values < 0.05.

## 3. Results

A total of 189 second-trimester anomaly scans were performed in the designated period and, after applying the exclusion criteria, 138 questionnaire responses were retained. The range of participants’ age was 17 to 50 years (mean = 30.3). Most participants (76.8%) had attained higher education (i.e., a university degree), while 21.7% had graduated from high school, and 1.4% graduated from primary school. With respect to marital status, 3% were unmarried. Further, in terms of the number of previous births, 75.4% were primiparas, 19.6% had previously given birth once, and 5% were multiparous. Only two participants declared having previous babies affected with congenital anomalies.

In 83.3% of the cases, the ob-gyn practitioner was the source of information about anomaly scanning, but it was verified by the internet and the participant’s social circle in 41.5 and 11% of the cases, respectively. The internet and social circle sources alone were relied upon by 11.6 and 5.1% of the participants, respectively.

Questions regarding the risks and limits of the ultrasound revealed that approximately one-third of the participants (32.6%) believed that the anomaly scan could detect all fetus defects, 13.8% believed that the fetus is bothered by the “light” emitted by the probe, and 8.7% were convinced that the ultrasound could harm the fetus.

The questions aimed at assessing the social and entertainment dimensions of the anomaly scan indicated that most of the participants (76.1%) were accompanied to the examination appointment by their husband, relatives, or friends; 96.4% declared that they would review the images and registrations with family and friends; and 10.1% intended to upload the images on social media.

**Figure 1:**
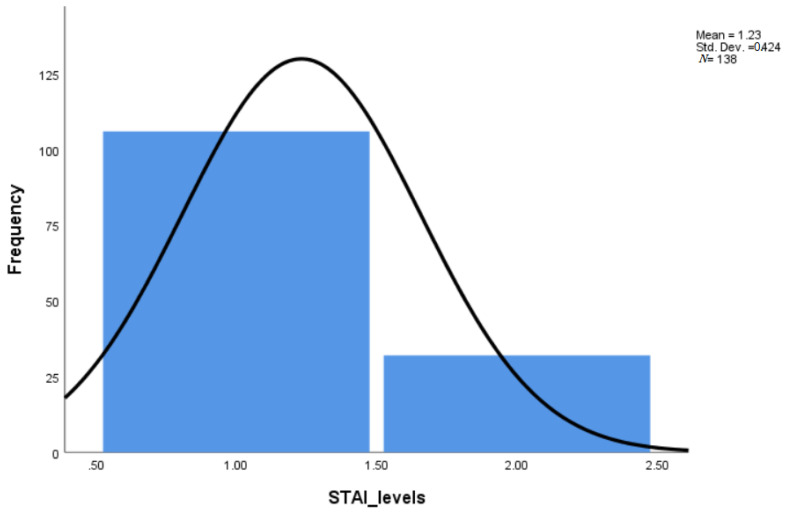
The pre-scan anxiety scores.

**Figure 2:**
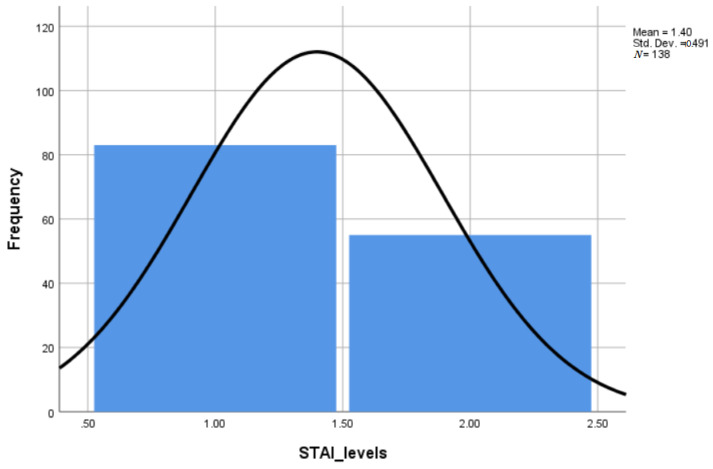
The post-scan anxiety scores.

Using the STAI score for pre-scan anxiety items, it was observed that 23.2% of the participants had high anxiety (Table 1, Figure 1 and Figure 2). The mean pre-scan anxiety score of the group was 9.52 (range = 6 to 21), corresponding to a low level of anxiety. Age was found to be positively related to pre-scan anxiety (*p* = 0.004).

The STAI score as a measure of the actual state of anxiety indicated that 39.9% of the participants were very anxious. The mean STAI score of the group was 38.4 (range = 20 to 76), corresponding to the upper limit of a low level STAT score. The correlation between pre-scan anxiety and STAI Form Y-1 score was positive, indicating that women with high state anxiety were more anxious at pre-scan (*p* = 0.001). Furthermore, STAI scores indicated that women with higher education (*p* = 0.041) were more anxious.

The third section of the questionnaire that evaluated the participants’ satisfaction with the anomaly scan indicated that for 96.4% of the participants, the scan was a pleasant experience, 97.8% were satisfied with the information provided by the scan, 99.3% were happy with the pictures and clips of the fetus that they received, and 93.5% felt that the scan strengthened their bond with the fetus.

Further, the communication with the performing physician was satisfying in 89.9% of the cases, but 11.6% of the participants wished for more “baby pictures” and 12.3% hoped for more assurance that the baby is well (Table 2). Women who wished for the examiner to talk more were the most nervous (*p* = 0.001) and those who wanted more images were the most confused (*p* = 0.002). The most relaxed participants reported that they would not review the images later (*p* = 0.026) and the mother–baby bond was stronger after the scan for the tensed participants (*p* = 0.003).

## 4. Discussion

Anxiety can be related to various aspects of a pregnancy, such as changes in the woman’s body, need for familial and social adaptation, prenatal procedures and investigations, and preparation for the process of birth. Anxiety is, therefore, frequently encountered in perinatal population and is related to postpartum depression [8,9,10].

Parentality requires a transformation process involving a couple, but each parent with their individual emotions and responses. In this process, the bonding between the mother and the baby is important in preparing the former to accept her role and the changes that will come with it [6]. Maternal-fetal bonding is a secondary but important effect of the ultrasound, especially if the scanning facilities provide 3D/4D images and clips [11]. In our study, more than 93% of the respondents acknowledged that seeing the fetus during the scan contributed to strengthening their bond with the fetus. This could perhaps be due to the visual effect of the fetal face and movements that bring the fetus to life (although virtually) and transforms the unseen almost abstract notion of the baby to a present and more real one [12,13].

In some women, the impact of recognizing the fetal facial traits as more or less similar to their parents or siblings also creates a powerful bond with the family members attending the ultrasound scan.

On the other hand, there can be also a negative impact of the fetal visualization and perception as “a real baby” if there is bad news or anomalies are found. Several studies demonstrate that “seeing the fetus” can make the decision to terminate an affected pregnancy more difficult [14]. These psychological effects have been discussed by Petchesky (1987) and used in antiabortion policies and materials such as in the movie “The Silent Scream”. This effect can also contribute to the maternal anxiety concerning the ultrasound [15].

Prenatal diagnosis is an additional source of stress for women due to the primary aim of the anomaly scan, that is, to reassure that the fetus is well and there is no reason for concern. Couples seek information about the baby’s health and wellbeing, but they are anxious about the possibility of finding an anomaly.

Such a finding raises several issues, such as concern about its implication on the baby’s health and life, the possibility and costs of treatment, the degree of dependence and handicap that could be involved, and the frightening eventuality that a pregnancy termination may have to be considered [16].

In our study, the scan anomaly-related anxiety was found to be moderate, and there may be several reasons for this. Most of the participants had a high level education and an urban background that allowed them access to information and medical assistance and also the possibility to have a better understanding of the significance of prenatal procedures. Conversely, access to unauthorized commonly available information and easy access could contribute to the anxiety towards the anomaly scan. As stated previously, approximately half of the responders doubled-checked medical information with other sources and 11.6% of the respondents relied solely on the internet, indicating a high degree of trust on sources of information that are not completely reliable.

Another issue specific to Romania is the lack of consistent information about what could be done in the eventuality of a fetal anomaly. This can impact the degree of maternal anxiety related to the anomaly scan. In Romania, protocols in case of fetal malformations do not exist and the counseling and management of pregnancy in such cases can be challenging for both patients and physicians [17]. The pregnancy termination provision whenever a fetal anomaly is in question is found only tangentially in Criminal Code and due to the inconsistencies there is room for local interpretation and practices under the menace of malpraxis and legal litigation. Neither the couples nor the physicians can rely on legal provisions in order to decide which fetal conditions permit termination after 14 weeks. Additionally, normative institutional psychological support is absent and in many cases, couples and physicians are on their own [18,19]. The response to the questionnaire item, “I am worried about what I could do in the eventuality of an anomaly” indicates that most patients are aware of and concerned about the incertitude and the psychological turmoil brought on by a fetal anomaly and that they rely mostly on family support rather than a professional psychotherapist.

Perinatal anxiety evaluated by means of anxiety measurement scales, such as STAI, was addressed by Meades et al. (2011) who reviewed their standardization in a perinatal population [20]. They reported that the measurement tools designed for the general population could not be applied to perinatal populations considering the various factors that can cause anxiety during this period (for instance, physical symptoms due to hormonal changes). On the other hand, identification of anxiety during the prenatal period can help the woman to develop coping mechanisms and prevent depressive disorders in the postpartum period. Women can learn cognitive and behavioral mechanisms to manage stressful situations such as a fetal anomaly diagnosis, to master emotions, and to make decisions. Recommendations for prenatal screening to aid early identification and prevention of postnatal depression dates back more than two decades, and in 1991, Levin conceptualized the Pregnancy Anxiety Scale, which is yet to be validated for different prenatal populations and procedures [21].

There are prenatal anxiety scales that have been designed for both ultrasound and invasive procedures, such as Košec et al.’s (2014) Prenatal Diagnostic Procedures Anxiety Scale (PDPAS) [22]. The scale requires validation with other populations, in order to assess the stress of procedure related to prenatal anxiety in correlation with specific characteristics of the investigated population and advancements of the ultrasound techniques. This is the reason why we did not utilize the scale and decided to construct a questionnaire comprising the first part of STAI and specific questions related to the ultrasound.

The entertainment dimension of the fetal ultrasound, and of particular interest, the second trimester anomaly scan, was explored in order to assess the risks versus benefits mainly in the countries (USA) where ultrasounds with an aim for commercial entertainment are permitted [23]. We found that in our sample, the ultrasound is also awaited with eagerness in consideration of the possibility to see the fetal face and to go home with beautiful pictures and even “videos”. This aspect of the fetal examination is imbedded in the Romanian women’s conscience to such an extent that a majority of them refer to the anomaly scan as “the 3D/4D scan”. A total of 96.4% of the respondents confirmed that they had similar expectations from the ultrasound and that the received images will be reviewed after the examination alone or with the family. In fact, for many couples, this aspect is of great importance, second only to the desire to get reassurance about fetal health, and the perfect picture and perfect fetus have lately seemed to induce a “rush”. There have been cases of expecting parents asking for a re-examination and exhibiting disappointment if for technical reasons obtaining 3D pictures is not possible. Overall, our results demonstrated that approximately 90% of the participants refrained from posting images of the fetus on social media platforms.

The main limitations of this study were the use of non-standardized questionnaire and the small sample size as there was some reluctance in filling such questionnaires both in the women and the physicians. The questionnaire had some sections being non-standardized beside the STAI. We could not find any validated and standardized questionnaire validated by our National College of Psychologists other than STAI and applicable to this topic. Further, the relatively large number of participants with high education and urban background may have biased the responses. However, the anomaly scan, which requires a third degree-certified sonographer is available mostly in big city clinics and, therefore, the majority of participants were from urban areas. A follow up of the participants would have been an asset to evaluate the predictive value of our questionnaires for mental health-related issues in late pregnancy or postpartum. The identification of a consistent correlation between high anxiety at the moment of anomaly scan and behavioral deviation in late pregnancy or postpartum depression would help in preventing or reducing the risks through attending and counseling the concerned women.

## 5. Conclusions

In the Romanian population, prenatal anomaly scan is a milestone in the pregnancy follow up for women and physicians, and this considerable importance comes along with both anxiety and preoccupation with the news that it may bring. Ultrasound scan in the second trimester is correlated with a significant anxiety for women who are prone to that psychological trait the time of scanning is a good opportunity to screen for high-risk women who are very anxious and who could benefit from prenatal psychological counseling for early detection and prevention of postpartum psychiatric disorders such as depression.

Further research is needed to extend the psychological evaluation to larger samples of pregnant women as well as to other prenatal procedures, considering that so far this has been a largely unexplored field in Romania.

## Figures and Tables

**Table 1:** Anxiety scores.

Pre-Scan Anxiety (STAI Pre-Scan)			Actual State of Anxiety (STAI Post-Scan)	
Frequency (Number)		Percent (%)	Frequency (Number)	Percent (%)
low	106	76.8	83	60.1
high	32	23.2	55	39.9
Total	138	100.0	138	100.0

**Table 2:** Patients characteristics.

Topic	Yes	No
Purpose of the ultrasound well explained	93.5%	6.5%
The couple will review the images	96.4%	3.6%
Distribution of the images on social networks	10.1%	89.9%
The ultrasound was pleasant	96.4%	3.6%
The knowledge that ultrasound detects fetal anomalies	32.6%	67.4%
The baby can be disturbed by light	13.8%	86.2%
The ultrasounds can be harmful for the baby	8.7%	91.3%
The information is interesting	97.8%	2.2%
The ultrasound is satisfactory	99.3%	0.7%
The ultrasound contributes to mother–baby bond	93.5%	5.8%
The physician should talk more	10.1%	89.9%
More images with the baby	11.6%	88.4%
More assurances about baby’s health	12.3%	87.7%

## Data Availability

The datasets used and/or analyzed during the current study are available from the corresponding author on reasonable request.

## Appendix A

This is a study which evaluates pregnant woman perceptions and feelings towards the ultrasound anomaly scan. Your participation is voluntary and anonym (no identification data are required) and by filling it you express your consent to participate in the study. The questionnaire has a part that must be completed before the scan and one that will give to you after the examination. Please take your time to read the questions and mark with an “X” the answer that you consider appropriate. The section that includes questions about feelings and emotions are included in two sections. There is no wrong or right answer. Please give the answer that you consider as describing your present stay or mood without thinking too much. Thank you for your kind cooperation.

Questions before the ultrasound scan.

Age			
Education (last attended)	Primary	High school	College
Marital status	married		other
How many children do you have?			
Do you have children with congenital anomalies?	Yes		NO
How did you get information about the scan?	Ob Gyn	Family, social circle	Internet
Did you get explanation about the aim of the ultrasound?	Yes		NO
According to your knowledge, can the scan detect all the baby’s anomalies	Yes		NO
Is the baby bothered by the scan light?	Yes		NO
According to your knowledge does the ultrasound harm the fetus?	Yes		NO
Are you accompanied at the examination?	Yes		NO
Will you review the pictures or “movies” with family and/or friends?	Yes		NO
Do you intend to broadcast the baby’s pictures on the internet? (Facebook, Tweeter, Instagram, other social media).	Yes		NO

Please mark the intensity of your feelings.

What are your feelings at the moment?	Not at all—1	Somewhat—2	Moderately so—3	Much so—4
I am afraid about the anomaly scan				
I feel strained because I had to wait until the anomaly scan				
I am afraid that the scan could harm the baby				
I am afraid about the possible anomalies that the scan can find				
I am concerned by the possible problems that the scan can find in me				
I am worried about what could I do in the eventuality of an anomaly				

Please mark the intensity of your current stay:

How do you feel?		Not at all	Somewhat	Moderately so	Much so
1.	I feel calm				
2.	I feel safe				
3.	I feel tense				
4.	I feel strained				
5.	I feel at ease				
6.	I feel angry				
7.	I am worried about possible problems				
8.	I feel satisfied				
9.	I feel jittery				
10.	I feel good				
11.	I feel self-confident				
12.	I feel nervous				
13.	I feel frightened				
14.	I feel indecisive				
15.	I feel relaxed				
16.	I feel content				
17.	I feel worried				
18.	I feel confused				
19.	I feel determined				
20.	I am pleasant				

Questions after the scan.

	Yes	No
Was the scan a pleasant experience?		
Did you get the information you wanted about the baby?		
The pictures received were satisfying		
I feel more connected with the baby after the ultrasound		
What would you liked during the scan:		
That the doctor talk more		
To get more pictures		
To get more insurances about the baby		
